# Development and Validation of an Algorithm for Constructing an Amino Acid Database for Application to the Korean Genome and Epidemiology Study Cohort

**DOI:** 10.3390/nu18071147

**Published:** 2026-04-02

**Authors:** Su-Jin Lee, Ji-Yun Hwang

**Affiliations:** 1 Department of Foodservice Management and Nutrition, Graduate School, Sangmyung University, Seoul 03016, Republic of Korea; juliasuj@naver.com; 2 Major of Foodservice Management and Nutrition, Sangmyung University, Seoul 03016, Republic of Korea

**Keywords:** amino acids, databases, algorithms, cohort studies, FFQ

## Abstract

Background/Objectives: The Korean Genome and Epidemiology Study (KoGES) is a large population-based cohort designed to investigate chronic disease risk using long-term dietary and health data. However, comprehensive amino acid information for estimating long-term intake from food frequency questionnaire (FFQ) data remains limited. This study aimed to develop and validate a standardized, rule-based algorithm for food matching and substitution and to construct an amino acid database applicable to the KoGES FFQ. Methods: The algorithm sequentially evaluated food name concordance, preparation forms, substitutability of similar foods, and differences in energy, macronutrients, and moisture (±20%). Amino acid composition data were derived from domestic and international food composition tables and published literature, with protein–nitrogen conversion factors applied by food group. Results: Amino acid information was established for 475 FFQ food items covering 19 amino acids. Of the database values, 31.0% were analytical, 64.2% were calculated, and 4.8% were substituted. Overall database coverage across all amino acid–food item combinations was 98.8%. The constructed database was applied to dietary data from the second follow-up (Phase 3) of the KoGES Ansan and Ansung community-based cohorts, showing that total amino acid intake accounted for 86.7% of total protein intake, reflecting the inclusion of non-protein nitrogen in conventional protein estimates. Based on the Estimated Average Requirement (EAR) criteria, the proportions of participants with intakes below the EAR for protein and essential amino acids varied across age and sex groups. Overall and in both men and women, lysine showed the highest proportion of participants below the EAR, whereas tryptophan showed the lowest proportion. Conclusions: This standardized algorithm provides a reproducible framework for constructing amino acid databases and can be applied to large-scale cohort and dietary survey data.

## 1. Introduction

Amino acids are the fundamental building blocks of proteins and play essential roles in numerous physiological processes, including protein synthesis, energy metabolism, immune function, and cellular signaling [1]. Beyond their structural roles, individual amino acids have distinct metabolic functions and are involved in various physiological processes related to health and disease [2,3]. Accordingly, growing evidence suggests that the quantity and composition of dietary amino acids, rather than total protein intake alone, are associated with chronic disease risk, metabolic health, and aging-related outcomes [4,5,6].

Accurate assessment of dietary amino acid intake is therefore essential in nutritional epidemiology. However, estimating amino acid intake at the population level remains methodologically challenging. Unlike energy or macronutrients, amino acid composition data are not comprehensively available for all foods, and protein values reported in food composition tables are often calculated using nitrogen conversion factors [7]. As a result, total protein intake alone may be insufficient to accurately characterize dietary protein quality, as differences in individual amino acid composition and digestibility can influence the nutritional contribution of protein-containing foods [8].

The construction of an amino acid database is a critical step in enabling amino-acid-specific dietary assessment. Such databases allow researchers to translate food intake data into quantitative estimates of individual amino acid intake, thereby facilitating more detailed analyses of diet–disease relationships [9]. This need is particularly pronounced in large-scale epidemiological studies that aim to evaluate long-term dietary exposure, where systematic errors in nutrient estimation can influence observed associations [10].

Early studies demonstrated the feasibility of estimating dietary amino acid intake through the development of amino acid composition databases linked to dietary intake data [9]. More recent research has expanded this approach by integrating nationally representative dietary survey data with comprehensive food composition databases to estimate population-level amino acid intake and examine temporal trends and major food sources [11,12]. Despite these advances, relatively few studies have focused on the development of standardized, rule-based algorithms for systematically assigning amino acid composition data to dietary intake instruments, particularly in the context of food matching, substitution, and the treatment of incomplete amino acid composition data.

The Korean Genome and Epidemiology Study (KoGES) is a large, population-based cohort designed to investigate the incidence and determinants of chronic diseases through comprehensive assessments of dietary intake, lifestyle factors, clinical indicators, and genetic information. Although the KoGES food composition database has been periodically updated and partial amino acid information has been incorporated, the application of a standardized, reproducible, algorithm-based framework for systematically estimating amino acid intake from food frequency questionnaire (FFQ) data, as well as consistent coverage of a broad range of amino acids, has not yet been fully established.

Therefore, the present study aimed to develop and validate a standardized, rule-based algorithm for food matching and substitution of amino acid composition data based on the KoGES FFQ. Using this algorithm, we constructed a comprehensive amino acid database and evaluated its coverage and applicability by applying it to data from the KoGES Ansan and Ansung cohorts. This study provides a transparent and reproducible methodological framework for amino-acid-specific dietary assessment in large-scale cohort and dietary survey research.

## 2. Materials and Methods

### 2.1. Study Population and Dietary Assessment

The KoGES is a large, population-based cohort established to investigate the incidence and determinants of chronic diseases among Korean adults. The present study used data from the Ansan and Ansung cohorts, applying dietary information collected during the second follow-up (Phase 3) survey conducted in 2005–2006 to examine the applicability of the constructed amino acid database in cohort-based dietary assessment. This survey wave was selected because it was the first assessment to employ the expanded 106-item FFQ, which was revised in 2004 to improve dietary coverage compared with the original 103-item FFQ developed in 2001 [13].

Dietary intake was assessed using a validated semi-quantitative FFQ developed for KoGES. The second follow-up survey employed the 106-item FFQ, designed to assess usual dietary intake over the previous year. Amino acid intake estimation was conducted based on 475 food items derived from the KoGES FFQ recipe database, to which the constructed amino acid database was applied. This consent form in the study was exempted by the Institutional Review Board of Sangmyung University (IRB-SMU-S2024-1-005), in accordance with the Bioethics and Safety Act, as it involved the secondary analysis of de-identified data.

### 2.2. Algorithm for Food Matching and Substitution in Amino Acid Database Construction

An amino acid database was constructed to enable the estimation of dietary amino acid intake from the KoGES food frequency questionnaire (FFQ). A standardized, rule-based algorithm was applied to assign amino acid composition values to FFQ food items through a sequential decision process. The rule-based algorithm was developed based on established procedures used in food composition database construction and previous studies on nutrient matching and substitution, with adaptations to reflect the structure and characteristics of the KoGES FFQ [9,11,12,14,15].

First, FFQ food items were matched to food composition database entries based on exact concordance of food names, which served as an initial screening criterion. When food names were concordant, the consistency of preparation and processing forms (e.g., raw, boiled, fried, or dried) was examined, as differences in preparation methods may substantially affect nutrient composition [9,14]. For food items with identical preparation forms, nutritional similarity was further evaluated by comparing energy, carbohydrate, protein, fat, and moisture contents. If differences in these components were within ±20%, analytically measured amino acid values were directly assigned to the corresponding FFQ food item [9,14,15]. The ±20% threshold was selected based on previous studies and established practices in food composition database matching, which consider this range acceptable for identifying nutritionally comparable foods while accounting for variability due to food composition, preparation methods, and analytical measurement [14].

If differences in energy or macronutrient composition exceeded ±20% despite identical preparation forms, the algorithm evaluated the availability of analytically measured amino acid data for nutritionally similar foods with different preparation forms. When such data were available, amino acid values were estimated using calculated values derived from analytically measured data of similar foods, in accordance with the predefined algorithmic rules. When neither directly matched analytical values nor analytically measured values from comparable foods were available, the assignment of amino acid values for the corresponding FFQ food item was temporarily deferred at this stage of the database construction process. Food items with incomplete or inconsistent compositional data were handled according to the predefined rule-based algorithm, which applies stepwise criteria for food matching, substitution, and calculation. This approach was intended to use the best available data while maintaining consistency in the assignment process.

When food names were not concordant, or when food names were concordant but preparation or processing forms were not identical, the algorithm next evaluated the availability of analytically measured amino acid data for similar food items. If analytical data for a similar food item were available and differences in energy, carbohydrate, protein, fat, and moisture contents were within ±20%, the corresponding amino acid values were directly assigned and classified as substituted values.

If analytical data for a similar food item were available but nutrient differences exceeded ± 20%, amino acid values were estimated using calculated values derived from the analytical data of the similar food item, in accordance with the algorithm-defined decision rules. If no analytically measured amino acid data were available for any similar food item, amino acid database construction for the corresponding FFQ food item was temporarily deferred.

In this algorithm, similar foods were defined based on food characteristics and data availability. For natural foods, similarity was determined by comparing taxonomic characteristics (species, genus, or family) using domestic and international analytical data. For processed foods, similarity was determined based on comparable food types, with consideration of the primary ingredient and its protein content.

### 2.3. Definition and Calculation of Total and Essential Amino Acids

Total amino acids (TAA) and essential amino acids (EAA) were calculated based on the definition and composition criteria adopted in the Korean Food Composition Table provided by the Rural Development Administration (RDA). Essential amino acids are defined as amino acids that cannot be synthesized endogenously in sufficient amounts and must therefore be obtained through the diet, as described in established nutritional guidelines. This approach was adopted because the Korean Food Composition Table represents the national standard for nutrient composition data and provided the highest proportion of available amino acid information among the data sources used in this study. Specifically, TAA was defined as the sum of the following 19 amino acids: isoleucine, leucine, lysine, methionine, phenylalanine, threonine, tryptophan, valine, histidine, arginine, tyrosine, cysteine, alanine, aspartic acid, glutamic acid, glycine, proline, and serine. EAA was defined as the sum of isoleucine, leucine, lysine, methionine, phenylalanine, threonine, tryptophan, valine, histidine, and arginine, consistent with the RDA classification, which includes histidine and arginine as essential amino acids during infancy and early growth; this classification was retained for consistency with the data source. For each FFQ food item, TAA and EAA were calculated by summing the available individual amino acid values. All individual amino acids, as well as TAA and EAA, were expressed in milligrams (mg), following the unit conventions of the Korean Food Composition Table.

When composition data for one or more constituent amino acids were unavailable for a given food item, TAA and EAA were computed using the sum of the remaining available amino acids, following the approach applied in the Korean Food Composition Table. As a result, TAA and EAA values were successfully derived for all 475 FFQ food items, yielding complete coverage for both indices despite partial missingness in specific amino acids such as taurine.

### 2.4. Amino Acid Data Sources and Nitrogen-Based Estimation

Amino acid composition data were obtained from multiple domestic and international sources, including the Korean Food Composition Table (10th, 10.3rd revisions) [16,17], the Standard Marine Products Composition Table (2018) [18], the Standard Processed Seafood Composition Table (2023) [19], the Standard Tables of Food Composition in Japan (7th revised edition) [20], the USDA FoodData Central database [21], and relevant published literature [22,23,24,25,26]. When multiple data sources were available for a given food item, analytically measured values from domestic databases were prioritized.

For food items without direct amino acid composition data, nitrogen content was derived from protein values using food-group-specific nitrogen-to-protein conversion factors. Nitrogen content was calculated as shown in Equation (1):

(1) $$N=\;\frac{\mathrm{Protein}\;\mathrm{content}\;(g/100\;g)}{\mathrm{Nitrogen}\text{-}\mathrm{to}\text{-}\mathrm{protein}\;\mathrm{conversion}\;\mathrm{factor}}$$

The nitrogen-to-protein conversion factors applied in this study were based on those used in the 9th revision of the Korean Food Composition Table, which were derived from the Standard Tables of Food Composition in Japan (7th revised edition). When amino acid values were estimated by substitution, a nitrogen correction approach was applied using the following Equation (2):

(2) $$\;\mathrm{Amino}\;\mathrm{acid}\;\mathrm{target}\;=\;\mathrm{Amino}\;\mathrm{acid}\;\mathrm{content}\;\mathrm{of}\;\mathrm{similar}\;\mathrm{food}\;\times \;\frac{\mathrm{Nitrogen}\;\mathrm{content}\;\mathrm{of}\;\mathrm{target}\;\mathrm{food}}{\mathrm{Nitrogen}\;\mathrm{content}\;\mathrm{of}\;\mathrm{similar}\;\mathrm{food}}$$

For natural foods, substitution was performed based on domestic analytical data with consideration of species and cultivar similarity. For processed foods, substitution was conducted using nutritionally similar food types, with additional consideration of nitrogen content and processing characteristics.

### 2.5. Database Quality Control and Application

Assigned amino acid values were classified into three categories according to their derivation: analytically measured values, calculated values, and substituted values. Coverage rates were calculated for each amino acid to assess database completeness, and all procedures were reviewed to minimize potential misclassification.

The constructed amino acid database was applied to dietary data from the KoGES Ansan and Ansung cohorts to estimate individual-level protein and amino acid intakes. Dietary intakes were assessed using a validated semi-quantitative FFQ, and amino acid intakes were calculated by linking FFQ food items to the constructed database. Total amino acid intake was defined as the sum of amino acids from all reported FFQ food items, while EAA intake was calculated as the sum of individual essential amino acids.

Participants were categorized into three age groups (30–49, 50–64, and 65–74 years) based on the Dietary Reference Intakes for Koreans (KDRI) age categories. Although the KDRI defines additional age groups, only these three categories were applicable to the age range of participants included in the present cohort analysis. Intake adequacy was evaluated by calculating the proportion of participants with protein and essential amino acid intakes below the Estimated Average Requirement (EAR), stratified by sex and age group. The use of EAR values from the KDRIs is appropriate for evaluating nutrient adequacy at the population level in generally healthy Korean adults, such as those included in the KoGES cohort, as EAR is specifically designed for group-level assessment. The validity of the constructed database was assessed by examining coverage rates and the consistency of the estimated amino acid intakes across participants. In addition, the relationship between total amino acid intake and protein intake was evaluated to assess internal consistency, and the distribution of amino acid intakes was compared with ranges reported in previous studies. Given that the protein EAR for Koreans aged 1 year and older is calculated on a body weight basis (0.66 g/kg/day)/0.9 × body weight, with additional allowances for growth during periods of development, the higher prevalence of intakes below the EAR among older adults and females suggests that protein intake per kilogram of body weight tends to be lower with increasing age and in females [27].

### 2.6. Statistical Analysis

Descriptive statistics were used to summarize the composition and coverage of the amino acid database. Amino acid intake estimates are presented as means and standard deviations. All statistical analyses were performed using SAS Studio (SAS version 9.4; SAS Institute Inc., Cary, NC, USA) via remote secure access provided by the Clinical & Omics Data Archive (CODA) at the National Institute of Health, Korea. Individual-level KoGES data were accessed and analyzed within the CODA research environment under approved data use agreements, ensuring secure data handling in accordance with institutional and national guidelines.

## 3. Results

**Figure 1:**
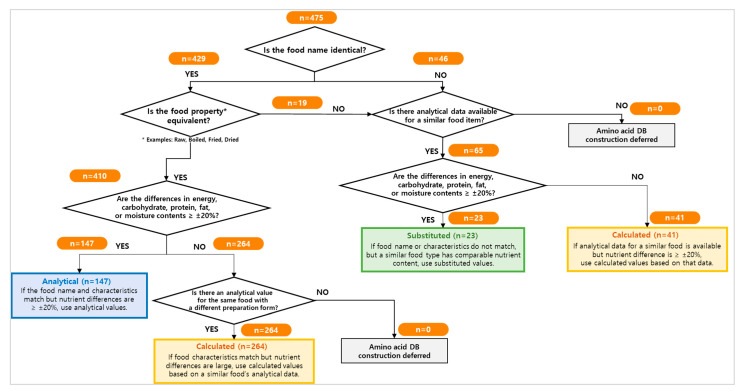
Flowchart of the rule-based algorithm for assigning amino acid values to FFQ food items. Analytical values were assigned when food name, preparation method, and nutrient composition differences were within ±20%. Substituted values were assigned when similar foods met the criteria, and calculated values were used when analytical data were unavailable or nutrient differences exceeded ±20%. The numbers (*n*) indicate the number of food items at each step. FFQ, food frequency questionnaire.

The developed rule-based algorithm was applied to all 475 food items included in the KoGES FFQ to evaluate its applicability and to quantify how food items were processed at each decision step. Figure 1 illustrates the distribution of FFQ food items as they progressed through the algorithm, with the number of items assigned at each decision node indicated.

Among the 475 FFQ food items, the majority were assigned amino acid values through direct matching or evaluation of equivalent food properties. A subset of food items required additional assessment based on nutrient differences and the availability of analytical data for similar foods, resulting in the assignment of calculated or substituted amino acid values. No FFQ food items were excluded from amino acid database construction at the final stage of the algorithm.

Table 1 summarizes the composition of the constructed amino acid database according to the type of assigned values and data sources for the 475 FFQ food items, in relation to the outcomes of the rule-based algorithm.

As shown in Figure 1, FFQ food items were first evaluated for the availability of analytically measured amino acid data and the presence of nutritionally comparable foods within the same food group. When foods with similar nutrient profiles were identified, amino acid values were assigned using either analytically measured values or substituted values derived from official food composition tables or validated analytical literature. Through this process, 30.9% (*n* = 147) of FFQ food items were assigned analytically measured values.

When foods with similar characteristics were available but showed substantial differences in energy, carbohydrate, protein, fat, or moisture content, amino acid values were assigned using calculated values based on nitrogen content and food-group-specific estimation procedures. This pathway accounted for the largest proportion of FFQ food items, 64.2% (*n* = 305), reflecting the frequent need for compositional adjustment during database construction. Calculated values were primarily derived from the Korean Food Composition Table (version 10.3), with supplementary data from official seafood composition tables, the Standard Tables of Food Composition in Japan, the USDA FoodData Central database, and peer-reviewed literature.

A smaller subset of FFQ food items, 4.8% (*n* = 23), required substituted values, which were assigned when analytically measured amino acid data were unavailable for the target food despite the presence of nutritionally similar foods. These substitutions were based on officially published food composition tables and were applied in accordance with the final decision steps of the algorithm.

In addition to national and international food composition tables, peer-reviewed analytical studies were used only for food items lacking complete amino acid profiles in official databases. Specifically, amino acid composition data for leaves of *Lactuca indica*, Japanese kelp (*Saccharina japonica*), wasabi leaves (*Wasabia japonica*), pinus leaves, and dog meat were obtained from studies employing officially recognized analytical methods. For dried kelp, overlapping analytical observations were available and incorporated into the database, resulting in a total of six analytical observations (*n* = 6).

Overall, the algorithm enabled the systematic assignment of amino acid values to all 475 FFQ food items (100%) by differentiating between analytical, calculated, and substituted values according to data availability and nutritional comparability, thereby ensuring comprehensive database coverage.

Table 2 summarizes the coverage of total and individual amino acids across the 475 FFQ food items included in the constructed amino acid database. Coverage was defined as the proportion of FFQ food items for which amino acid values were assigned using the rule-based algorithm. TAA and EAA were available for all FFQ food items, resulting in complete coverage. All individual essential amino acids, including isoleucine, leucine, lysine, methionine, phenylalanine, threonine, valine, and histidine, also showed full coverage. Among non-essential amino acids, most achieved complete coverage across FFQ food items. Tryptophan and tyrosine showed slightly lower coverage, with values assigned to 98.1% (*n* = 466) and 99.2% (*n* = 471) of FFQ food items, respectively. Taurine values were available for 77.1% (*n* = 366) of FFQ food items. Overall, the coverage varied across individual amino acids, reflecting differences in data availability among amino acid composition sources.

Dietary protein and amino acid intakes estimated by applying the constructed amino acid database to the KoGES Ansan and Ansung cohort data are presented in Table 3. When applied to cohort dietary data, the database produced distributions of protein and amino acid intakes that were comparable to previously reported ranges in similar adult populations indirectly supporting the applicability and validity of the constructed amino acid database.

Among individual essential amino acids, leucine (3903.7 mg/day) and lysine (2806.0 mg/day) showed relatively higher intakes, whereas tryptophan intake (579.4 mg/day) was the lowest. Intakes of all essential amino acids were higher in men than in women. Among non-essential amino acids, glutamic acid (9625.4 mg/day) and aspartic acid (4875.5 mg/day) accounted for the highest intakes, while taurine intake was 136.8 mg/day. Overall, most individual amino acid intakes were higher in men than in women.

**Figure 2:**
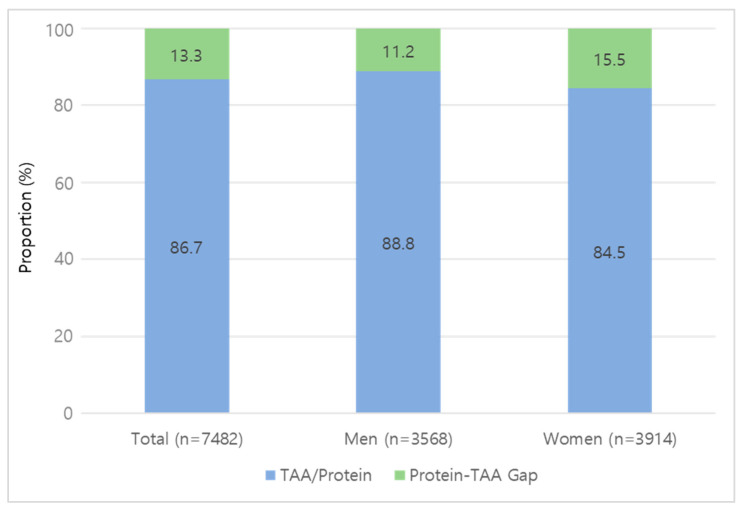
Proportion of total protein intake explained by TAA. TAA accounted for 86.7% of total protein intake overall, with corresponding values of 88.8% in men and 84.5% in women. The remaining proportion represents non-amino acid nitrogen. TAA, total amino acids.

The mean protein intake among all participants was 57.0 g/day, while TAA intake was corresponding to 86.7% of total protein intake (Figure 2). The mean intake of EAA was 21,703.4 mg/day. By sex, mean protein intake was 60.8 g/day in men and 53.5 g/day in women. The corresponding TAA intakes were 54,026.9 mg/day in men and 45,165.3 mg/day in women, representing 88.8% and 84.5% of protein intake, respectively (Figure 2). Mean EAA intake was 23,797.1 mg/day in men and 19,794.9 mg/day in women.

**Figure 3:**
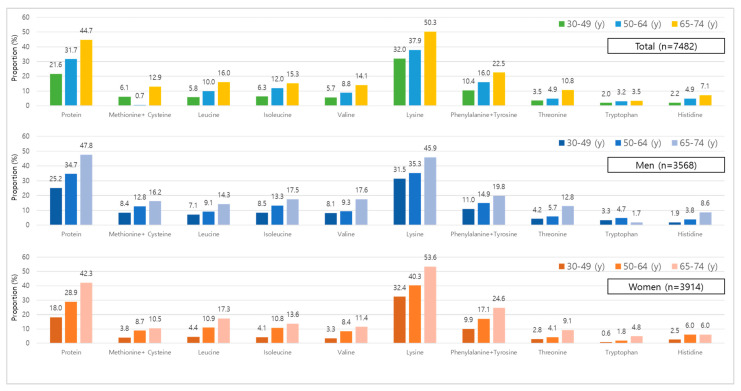
Proportion of participants below EAR for protein and essential amino acids, by sex. The top panel shows the total population, and the middle and bottom panels show men and women, respectively. Within each panel, bars represent age groups (30–49, 50–64, and 65–74 years). Values are expressed as percentages (%). EAR, Estimated Average Requirement.

The proportions of participants with intakes below the EAR for protein and essential amino acids by age group and sex are shown in Figure 3. In the total population, the prevalence of inadequate intake generally increased with age across most nutrients. Participants aged 65–74 years consistently exhibited the highest proportions of EAR inadequacy, followed by those aged 50–64 years and 30–49 years.

Protein inadequacy increased markedly with age, rising from 21.8% among adults aged 30–49 years to 44.7% in those aged 65–74 years. Similar age-related trends were observed for lysine (32.0% to 50.3%) and phenylalanine + tyrosine (10.4% to 22.5%). Branched-chain amino acids, including leucine, isoleucine, and valine, also showed progressively higher inadequacy rates in older age groups.

Sex-stratified analyses revealed that women consistently exhibited higher proportions of EAR inadequacy than men across most nutrients and age groups. Among women, the prevalence of protein intake below the EAR increased from 18.0% in the 30–49-year group to 42.3% in the 65–74-year group, whereas the corresponding values in men were 25.7% and 47.8%, respectively. Similar patterns were observed for lysine and other essential amino acids.

In contrast, tryptophan and histidine showed relatively low proportions of inadequacy across all age and sex groups, although older adults still demonstrated higher prevalence than younger adults. Overall, these findings indicate pronounced age-related increases in EAR inadequacy, with women generally exhibiting higher rates of inadequate protein and essential amino acid intakes compared with men.

## 4. Discussion

This study developed and implemented a standardized, rule-based algorithm to construct an amino acid database applicable to FFQ data and applied the resulting database to the KoGES Ansan and Ansung cohorts. The algorithm systematically integrated analytically measured, calculated, and substituted amino acid values using sequential decision rules based on food characteristics, preparation methods, and nutritional similarity, enabling transparent documentation of assignment pathways across all FFQ food items. Through this approach, amino acid values were successfully assigned to 100% of FFQ foods, achieving complete coverage for total amino acids and essential amino acids, with high coverage for most non-essential amino acids.

Application of the constructed database to the second follow-up (Phase 3) of the KoGES Ansan and Ansung community-based cohorts yielded plausible distributions of protein and amino acid intakes and enabled comprehensive population-level assessment of intake patterns and adequacy. Total amino acid intake accounted for approximately 86.7% of total protein intake, reflecting the expected relationship between protein and its constituent amino acids. Although the observed protein and amino acid intakes were lower than those reported in the Korea National Health and Nutrition Examination Survey [12,28], they were comparable to values reported in previous cohort-based studies, suggesting consistency with long-term dietary assessment in community-based populations [14]. Among essential amino acids, leucine and lysine contributed the largest shares of intake, whereas tryptophan showed the lowest intake, consistent with known amino acid composition patterns of commonly consumed foods. Moreover, intakes of most amino acids were higher in men than in women. Among non-essential amino acids, glutamic acid showed the highest intake, which is consistent with previous reports reflecting its abundance in protein-rich foods and mixed diets [12].

Importantly, the database facilitated evaluation of intake adequacy using EAR-based criteria, revealing substantial proportions of participants with inadequate intakes of protein and several essential amino acids. Pronounced age-related increases in EAR inadequacy were observed, with adults aged 65–74 years consistently exhibiting the highest prevalence, particularly for protein, lysine, phenylalanine + tyrosine, and branched-chain amino acids. Clear sex differences were also evident, with women generally showing higher proportions of EAR inadequacy across most nutrients and age groups. Together, these findings demonstrate that the constructed database not only supports estimation of dietary amino acid intake from FFQ data but also enables identification of nutritionally vulnerable subgroups within population-based cohorts. These observed disparities may be partly explained by differences in overall energy and protein intake across age and sex groups. Older adults may have lower total food intake, while women may have lower absolute protein intake compared to men, which could contribute to the higher prevalence of inadequacy observed among older adults and women, consistent with previous findings in Korean populations reporting age- and sex-related differences in nutrient intake and adequacy [29]. Furthermore, given that the protein EAR for Koreans aged 1 year and older is calculated on a body weight basis (0.66 g/kg/day)/0.9 × body weight, with additional allowances for growth during periods of development, the higher prevalence of intakes below the EAR among older adults and women suggests that protein intake per kilogram of body weight tends to be lower with increasing age and in women [27].

These observed patterns of amino acid inadequacy are particularly relevant given growing evidence linking dietary amino acid intake to metabolic health. Beyond methodological considerations, accumulating evidence indicates that dietary amino acid intake is associated with metabolic health and chronic disease outcomes. Previous studies in Korean adult populations have reported associations between amino acid intake patterns and cardiometabolic risk factors, including dyslipidemia, metabolic syndrome, and insulin resistance [3,6,30]. These findings underscore the relevance of amino-acid-specific dietary assessment beyond total protein intake and highlight the importance of reliable estimation of individual amino acid intake in epidemiological research.

A major strength of the present study lies in the use of a standardized, algorithm-based approach to guide amino acid value assignment from FFQ data. Dietary assessment in epidemiological research commonly relies on self-reported methods, such as FFQs, which are known to be subject to various sources of error, including limitations in food composition databases used to convert reported food intake into nutrient estimates [31]. In this context, careful handling of food matching and nutrient assignment procedures is essential to improve the quality and interpretability of dietary intake data.

Previous studies have demonstrated the feasibility of estimating dietary amino acid intake by linking food composition databases to dietary assessment data [9,11]. However, food composition data are often derived from multiple sources, and amino acid information may be incomplete or unavailable for certain foods, necessitating the use of calculated or substituted values. Methodological research on food composition data emphasizes that, when such procedures are applied, the criteria and decision processes should be clearly defined and documented [32].

In the present study, sequential decision rules were explicitly established to guide food matching, substitution, and estimation of amino acid values, considering food names, preparation methods, and nutritional similarity. By formalizing these procedures within a transparent framework, the algorithm-based approach adopted in this study enhances methodological clarity and reproducibility. Such a structured approach may support the application of amino acid database construction to other cohort studies and dietary assessment instruments, particularly in large-scale epidemiological settings where heterogeneous food composition data are commonly encountered [31,32].

Another notable feature of the constructed database is its comprehensive coverage across FFQ food items. Complete coverage was achieved for total amino acids and all EAAs, while most non-essential amino acids also showed full or near-complete coverage. These results reflect the effectiveness of the standardized algorithm in accommodating diverse food items and data sources. Similar challenges in achieving complete amino acid coverage have been reported in other amino acid database construction efforts; for example, a Japanese amino acid composition database contained values for only a fraction of foods in the standard table of food composition, necessitating extensive imputation for missing values [9].

Lower coverage was observed for certain amino acids such as taurine, reflecting the limited availability of analytically measured taurine composition data in existing food composition databases. This limitation is inherent to the nature of available food composition data rather than the database construction process itself and highlights the need for continued expansion of analytical data for specific amino acids in food composition research.

Several limitations of this study should be acknowledged. First, the constructed database did not include all amino acids uniformly across all food items, reflecting constraints in the availability of analytically measured amino acid composition data. Although the standardized algorithm enabled extensive coverage, incomplete data for certain amino acids remained an inherent limitation of current food composition resources. Second, the amino acid database developed in this study was designed and applied specifically for use with cohort-based dietary data derived from the KoGES FFQ. Accordingly, its direct applicability to other dietary assessment tools or survey formats may be limited, and caution is warranted when extending the database beyond similar cohort settings. In addition, as the database was developed based on food items, dietary patterns, and food composition data specific to the Korean population, its direct application to other populations may be limited. However, the overall framework of the rule-based algorithm, including the stepwise approach to food matching, substitution, and estimation, may be adaptable to other populations with appropriate modification of food composition databases and context-specific criteria.

Finally, the present database primarily focused on foods represented in the cohort FFQ, and comprehensive amino acid composition data for a wide range of processed foods were not constructed. In addition, the applicability of the database was evaluated using FFQ-based dietary data only, and direct validation against short-term dietary assessment methods, such as 24 h dietary recalls or weighed food records, was not performed. Future studies incorporating expanded processed food databases and multiple dietary assessment methods would strengthen the robustness and generalizability of amino acid intake estimation.

Despite these limitations, the present study provides a transparent and reproducible methodological framework for constructing amino acid databases applicable to epidemiological research using FFQ data. By explicitly defining sequential decision rules for food matching, substitution, and estimation, the algorithm-based approach developed in this study enables systematic handling of heterogeneous and incomplete amino acid composition data in large-scale cohort settings.

Given the growing body of evidence linking dietary amino acid intake to chronic disease risk in Korean populations [3,6,26], the constructed database and algorithmic framework offer a practical foundation for investigating amino acid–disease relationships beyond total protein intake. Furthermore, the successful application of the database to the KoGES Ansan and Ansung cohorts demonstrates its feasibility for population-level dietary assessment within long-term cohort studies. Future research may build upon this framework by incorporating additional analytically measured amino acid data, expanding coverage for underrepresented food groups—including processed foods—and applying the algorithm to other dietary assessment tools and population groups. Such efforts would further strengthen the validity, robustness, and generalizability of amino acid intake estimation in nutritional epidemiology.

## 5. Conclusions

This study developed a standardized, rule-based algorithm to construct an amino acid database applicable to FFQ data and successfully applied it to the second follow-up (Phase 3) of the KoGES Ansan and Ansung community-based cohorts. The algorithm enabled systematic assignment of amino acid values to all FFQ food items, achieving complete coverage for total and essential amino acids and high coverage for most non-essential amino acids. Application of the database yielded plausible distributions of dietary protein and amino acid intakes and enabled EAR-based evaluation of intake adequacy, identifying marked age- and sex-related disparities, particularly among older adults and women. Beyond supporting estimation of dietary amino acid intake, this transparent and reproducible framework provides a practical foundation for future investigations of amino acid–disease relationships and for improving amino acid intake assessment in large-scale nutritional epidemiology.

## Figures and Tables

**Table 1:** Amino Acid Data Sources for the FFQ-based Cohort Study.

Category	Data Source	No. of Items	%
Analytical values	Korean Food Composition Table ver. 10.3	137	28.8
	Korean Food Composition Table ver. 10.0	2	0.4
	Standard Table of Seafood Composition 2018 and 2023	8	1.7
	Subtotal	147	30.9
Calculated values	Korean Food Composition Table ver. 10.3	273	57.5
	Korean Food Composition Table ver. 10.0	1	0.2
	Standard Table of Seafood Composition 2018	21	4.4
	Standard Tables of Food Composition in Japan (7th ed.)	2	0.4
	USDA FoodData Central	2	0.4
	Literature search (published papers)	6	1.3
	Subtotal	305	64.2
Substituted values	Korean Food Composition Table ver. 10.3	21	4.4
	Standard Table of Seafood Composition 2018 and 2023	2	0.4
	Subtotal	23	4.8
Total		475	100.0

In the Standard Table of Seafood Composition 2023, only taurine content was referenced and utilized.

**Table 2:** Coverage of amino acid composition data across FFQ food items.

Variables	N of Food	Coverage of 475 Foods (%)
TAA	475	100.0
EAA	475	100.0
Isoleucine	475	100.0
Leucine	475	100.0
Lysine	475	100.0
Methionine	475	100.0
Phenylalanine	475	100.0
Threonine	475	100.0
Tryptophan	466	98.1
Valine	475	100.0
Histidine	475	100.0
Arginine	475	100.0
Tyrosine	471	99.2
Cysteine	475	100.0
Alanine	475	100.0
Aspartic acid	475	100.0
Glutamic acid	475	100.0
Glycine	475	100.0
Proline	475	100.0
Serine	475	100.0
Taurine	366	77.1
Overall coverage ^†^	-	98.8

TAA, total amino acids; EAA, essential amino acids. ^†^ Overall database coverage was calculated as the proportion of non-missing amino acid values across all amino acid–food item combinations (i.e., populated cells divided by the total possible cells), rather than by the number of food items.

**Table 3:** Dietary amino acid intake of participants in the KoGES Ansan and Ansung cohorts.

Variables	Total (*n* = 7482)		Men (*n* = 3568)		Women (*n* = 3914)	
	Mean	SE	Mean	SE	Mean	SE
Age	56.1	0.1	55.6	0.1	56.6	0.1
Energy (kcal)	1840.3	7.1	1965.1	9.8	1726.5	9.9
Protein (g)	57.0	0.3	60.8	0.4	53.5	0.4
TAA (mg)	49,391.2	320.9	54,026.9	446.9	45,165.3	448.2
EAA (mg)	21,703.4	151.0	23,797.1	211.3	19,794.9	210.4
Isoleucine (mg)	1859.6	14.2	2042.8	19.8	1692.6	19.8
Leucine (mg)	3903.7	26.1	4268.3	36.5	3571.4	36.4
Lysine (mg)	2806.0	24.0	3126.1	34.4	2514.3	32.8
Methionine (mg)	968.7	7.7	1072.2	10.8	874.2	10.8
Phenylalanine (mg)	2286.9	14.4	2479.5	19.6	2111.3	20.5
Threonine (mg)	2095.1	14.8	2298.4	20.8	1909.8	20.5
Tryptophan (mg)	579.4	3.8	627.9	5.4	535.2	5.3
Valine (mg)	2281.3	16.1	2492.3	22.2	2089.1	22.8
Histidine (mg)	1451.2	10.0	1598.7	13.8	1316.8	13.9
Arginine (mg)	3480.3	20.8	3799.1	29.2	3189.7	28.8
Tyrosine (mg)	1697.9	11.2	1851.3	15.7	1558.1	15.7
Cysteine (mg)	732.2	3.5	781.3	4.7	687.5	5.0
Alanine (mg)	2702.4	17.6	2971.2	24.8	2457.3	24.3
Aspartic acid (mg)	4875.5	30.7	5278.4	42.8	4508.2	42.9
Glutamic acid (mg)	9625.4	56.8	10,460.3	77.0	8864.3	81.0
Glycine (mg)	2471.3	16.4	2748.0	23.0	2219.0	22.6
Proline (mg)	2881.4	21.1	3215.3	31.4	2577.0	27.6
Serine (mg)	2588.5	14.6	2804.2	20.2	2391.8	20.6
Taurine (mg)	136.8	1.7	141.5	2.2	132.5	2.5

TAA, total amino acids; EAA, essential amino acids.

## Data Availability

The data used in this study were obtained from the Korean Genome and Epidemiology Study (KoGES). Due to privacy and ethical restrictions, the data are not publicly available but may be accessed from the Korea National Institute of Health upon reasonable request and approval.

## References

[1] Hyun T., Han S., Kim H., Kwon Y., Jung J. (2022). Plus Advanced Nutrition.

[2] Wu G. (2009). Amino acids: Metabolism, functions, and nutrition. Amino Acids.

[3] Chung S., Park J.H., Joung H., Ha K., Shin S. (2023). Amino acid intake with protein food source and incident dyslipidemia in Korean adults. Front. Nutr..

[4] Izadi N., Hadi R., Shafiee A., Fathi H., Niknam M., Amiri P. (2025). Dietary amino acid intake and the risk of hypertension: A systematic review and meta-analysis. BMC Public Health.

[5] Najafi F., Mohseni P., Pasdar Y., Niknam M., Izadi N. (2023). The association between dietary amino acid intake and risk of type 2 diabetes mellitus: A meta-analysis. PLoS ONE.

[6] Chae M., Park H.S., Park K. (2021). Association between dietary branched-chain amino acid intake and skeletal muscle mass index among Korean adults: Interaction with obesity. Nutr. Res. Pract..

[7] Mariotti F., Tomé D., Mirand P.P. (2008). Converting nitrogen into protein—Beyond 6.25 and Jones’ factors. Crit. Rev. Food Sci. Nutr..

[8] FAO (2013). Dietary Protein Quality Evaluation in Human Nutrition. FAO Food and Nutrition Paper No. 92.

[9] Suga H., Murakami K., Sasaki S. (2013). Development of an amino acid composition database and estimation of amino acid intake in Japanese adults. Asia Pac. J. Clin. Nutr..

[10] Freedman L.S., Commins J.M., Moler J.E., Arab L., Baer D.J., Kipnis V., Midthune D., Moshfegh A.J., Neuhouser M.L., Prentice R.L. (2014). Pooled results from 5 validation studies of dietary self-report instruments using recovery biomarkers for energy and protein intake. Am. J. Epidemiol..

[11] Tsumura A., Yamanaka-Okumura H., Kawakami H., Yamamoto S., Oura M., Ohminami H., Taketani Y. (2023). Amino acid and fatty acid profiles of the average Japanese diet: Fusion of the National Health and Nutrition Survey and the Food Composition Database. Hum. Nutr. Metab..

[12] Kim S., Ham H., Ha K. (2025). Trends in dietary amino acid intake and food sources among Korean adults: Data from the 2010–2022 Korea National Health and Nutrition Examination Survey. Nutr. Res. Pract..

[13] Ahn Y., Lee J.E., Paik H.Y., Lee H.K., Jo I. (2003). Development of a semiquantitative food frequency questionnaire based on dietary data from the Korea National Health and Nutrition Examination Survey. Nutr. Sci..

[14] Park S.J., Lyu J., Lee K., Lee H.J., Park H.Y. (2024). Nutrition survey methods and food composition database update of the Korean Genome and Epidemiology Study. Epidemiol. Health.

[15] Yoon M.O., Kim K., Hwang J.Y., Lee H.S., Son T.Y., Moon H.K., Shim J.E. (2014). Development of a fatty acids database using the Korea National Health and Nutrition Examination Survey data. J. Nutr. Health.

[16] Rural Development Administration (RDA) (2021). Standard Food Composition Table.

[17] Rural Development Administration (RDA) (2024). Standard Food Composition Table.

[18] National Institute of Fisheries Science (NIFS) (2018). Standard Marine Products Composition Table.

[19] National Institute of Fisheries Science (NIFS) (2023). Standard Processed Seafood Composition Table.

[20] Ministry of Education, Culture, Sports, Science and Technology (MEXT) (2015). Standard Tables of Food Composition in Japan—2015.

[21] U.S. Department of Agriculture, Agricultural Research Service (USDA-ARS) (2019). FoodData Central.

[22] Kim S.Y., Park K.C. (1997). Comparison of chemical constituents of upland *Wasabia japonica* Matsum grown by different propagation methods. Korean J. Med. Crop Sci..

[23] Jung H.-J. (2017). The Minerals Analysis and Nutritional Evaluation according to Production Area of Laver, Japanese Kelp, Sea Mustard, Hijiki in Korea (*Porphyra tenera*, *Saccharina japonica*, *Undaria pinnatifida*, *Sargassum fusiforme*). Master’s Thesis.

[24] Hwang B.-H., Cho J.-H., Ham S.-S., Kang H.-Y. (2000). Chemical analysis of *Pinus* leaves. J. Korean Soc. Food Sci. Nutr..

[25] Kim J.-M., Kim J.-N., Lee K.-S., Shin S.-R., Yoon K.-Y. (2012). Comparison of physicochemical properties of wild and cultivated *Lactuca indica*. J. Korean Soc. Food Sci. Nutr..

[26] Ann Y.-G. (1999). Dog meat foods in Korea. Korean J. Food Nutr..

[27] Ministry of Health and Welfare, The Korean Nutrition Society (2025). Dietary Reference Intakes for Koreans 2025 (English Summary Version).

[28] Kim C.I., Jang Y.A., Lee H.S., Lee E.Y., Lee H.J. (2005). The Third Korea National Health and Nutrition Examination Survey (KNHANES III), 2005: Nutrition Survey.

[29] Byeon W., Kim C.I., Kwon S.O., Yoon J., Huang L. (2025). Nutritional risk assessment using estimated usual nutrient intake in Korean adults: Analysis of the 8th (2019–2021) Korea National Health and Nutrition Examination Survey data. Nutr. Res. Pract..

[30] Im J., Park H., Park K. (2022). Higher Intake of Total Dietary Essential Amino Acids Is Associated with a Lower Prevalence of Metabolic Syndrome among Korean Adults. Nutrients.

[31] Pennington Jean A.T. (2008). Applications of food composition data: Data sources and considerations for use. J. Food Compos. Anal..

[32] Naska A., Lagiou A., Lagiou P. (2017). Dietary assessment methods in epidemiological research: Current state of the art and future prospects. F1000Research.

